# Design and Analysis of FBG Acceleration Sensor with Double-Sided Symmetrical Inclined Cantilever Beam Structure

**DOI:** 10.3390/s26144347

**Published:** 2026-07-09

**Authors:** Yuqi Tian, Mingpan Zou, Xuan Zhao, Pengyu Zhang, Hanbing Yan, Wenqing Wei, Zhixiang Wu, Hu Deng

**Affiliations:** 1 School of Information Engineering, Southwest University of Science and Technology, Mianyang 621010, China; tianyq@swust.edu.cn (Y.T.); denghu@swust.edu.cn (H.D.); 2 School of Automation, Chengdu University of Information Technology, Chengdu 610225, China; 3 Hangzhou Hikimaging Technology Co., Ltd., Hangzhou 310051, China; 4 Sichuan Provincial Engineering Research Center for Artificial Intelligence Applications in Transportation, Sichuan Vocational and Technical College of Communications, Chengdu 611130, China

**Keywords:** fiber Bragg grating, accelerometer, bilaterally symmetric tilted cantilever beam, high-frequency impact

## Abstract

To meet the demand for high-range and high-frequency impact acceleration measurements, a fiber Bragg grating (FBG) accelerometer with a double-sided symmetrical inclined cantilever beam structure is proposed. The sensing principle is analyzed based on inertial and FBG sensing theories, and theoretical models for sensitivity and resonant frequency are derived. Using PPA-CF material and parameter optimization, the structural dimensions of the cantilever beam, effective fiber length, and proof mass are determined through numerical simulation. Finite element modal analysis shows a first-order natural frequency of 1284 Hz, with the dominant mode being axial translation of the mass block, ensuring pure axial strain on the FBG. A drop-weight impact calibration system is established for experimental testing. Results demonstrate that the sensor achieves a resonant frequency exceeding 1333 Hz, a measurement range above 500 g, and a practical sensitivity of approximately 1.183 pm/g. The linear response exhibits a coefficient of determination, *R*^2^, of 0.9875, confirming excellent linearity. The proposed accelerometer effectively balances high-frequency response and measurement accuracy, providing a reliable solution for high-frequency impact monitoring in aerospace and impact dynamics applications.

## 1. Introduce

Acceleration measurement is a critical parameter in fields such as experimental mechanics, aerospace impact testing, and dynamic response monitoring in civil engineering. By acquiring acceleration data, it is possible to assess structural safety, material dynamic response, and disaster prediction, among other aspects. Although traditional electrical sensing technologies have reached a high level of maturity, as measurement environments become increasingly complex and measurement requirements continue to rise, electrical sensors—due to their inherent susceptibility to electromagnetic interference and electrical sparks—are struggling to meet the demands of increasingly demanding application scenarios. In contrast, fiber Bragg grating (FBG) accelerometers demonstrate irreplaceable application potential due to their superior characteristics, including resistance to electromagnetic interference, corrosion resistance, compact size, and ease of distributed networking [1,2]. However, because FBG accelerometers have been in development for a relatively short period, issues such as insufficient dynamic response and limited measurement range still exist in practical applications, which significantly restricts their applicability in engineering practice [3,4,5].

Although existing FBG accelerometers exhibit irreplaceable advantages in complex environments, they face a critical trade-off between dynamic response and measurement range. Conventional designs primarily utilize two configurations: the cantilever beam type, which suffers from low natural frequency [6,7,8], and the flexible hinge type, which risks structural damage under high impacts [9,10,11]. Beyond structural limitations, material selection further constrains performance; conventional metal alloys often introduce excessive mass, inevitably lowering the resonant frequency. To overcome these barriers, this study adopts high-stiffness, low-density PPA-CF composites to fabricate a bilaterally symmetric tilted cantilever beam structure. This design optimizes the stiffness-to-mass ratio required for high-frequency operation (>1000 Hz) and a wide measurement range (>500 g), while ensuring the FBG experiences pure axial strain to mitigate errors from transverse shear forces.

Regarding the overall structure of this paper, Section 1 first clarifies the research background and current status of accelerometers; Section 2 analyzes the operating principle and structure of the double-sided symmetrical tilted cantilever beam sensor; Section 3 optimizes the sensor’s structural parameters based on composite materials; and Section 4 verifies the sensor’s actual measurement performance through experiments. This paper aims to provide theoretical guidance for practical engineering applications through an in-depth exploration of grating accelerometers.

## 2. Working Principle of the Sensor

### 2.1. Principle of FBG Acceleration Sensor

**Figure 1:**
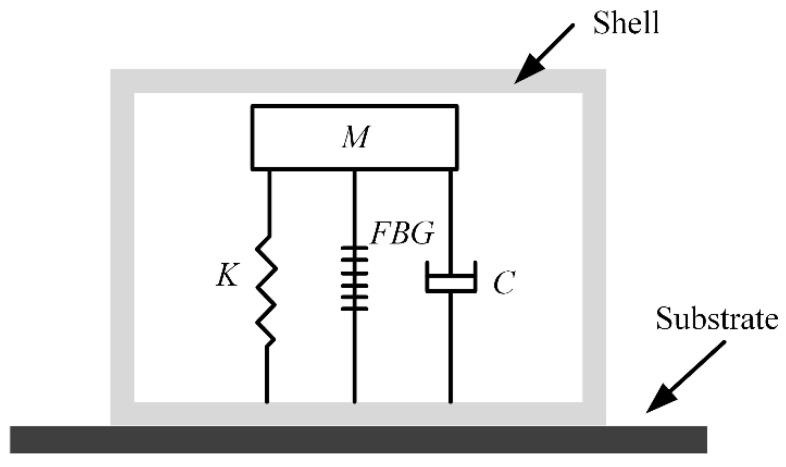
Mechanical model diagram of the FBG acceleration sensor.

The FBG accelerometer combines the principles of inertial measurement with those of fiber Bragg grating (FBG) sensing. It replaces the electrical sensing elements of traditional accelerometers with an FBG, converting the deformation of the elastic element into a shift in the center wavelength of the FBG. Acceleration is measured by detecting this wavelength shift. Its core structure consists of an inertial mass block M, an elastic element K, a damper C, and an FBG sensing unit. The mechanical model is shown in Figure 1. When the sensor is subjected to external vibration or impact, the inertial force generated by the inertial mass block causes the elastic element to deform. The FBG undergoes axial strain along with the elastic element, thereby causing a shift in the center wavelength.

The dynamic magnification factor *β* of this single-degree-of-freedom system is defined as the ratio of the steady-state forced vibration amplitude to the housing vibration amplitude, and its expression is:

(1) $$\beta =\frac{A}{x_{0}}=\frac{\gamma^{2}}{\sqrt{{\left(1-\gamma^{2}\right)}^{2}+{\left(2\xi \gamma \right)}^{2}}}$$

where *γ* = *ω*/*ω*_0_ is frequency ratio, *ω* is the angular frequency of the measured vibration, *ω*_0_ is the natural angular frequency of the system, *A* is the amplitude of the steady-state forced vibration of the mass block, *x*_0_ is the amplitude of the vibration of the housing relative to the ground, and *ξ* is the damping ratio of the system.

K represents the system’s stiffness, C represents the system’s damping coefficient, and M represents the mass of the mass block.

**Figure 2:**
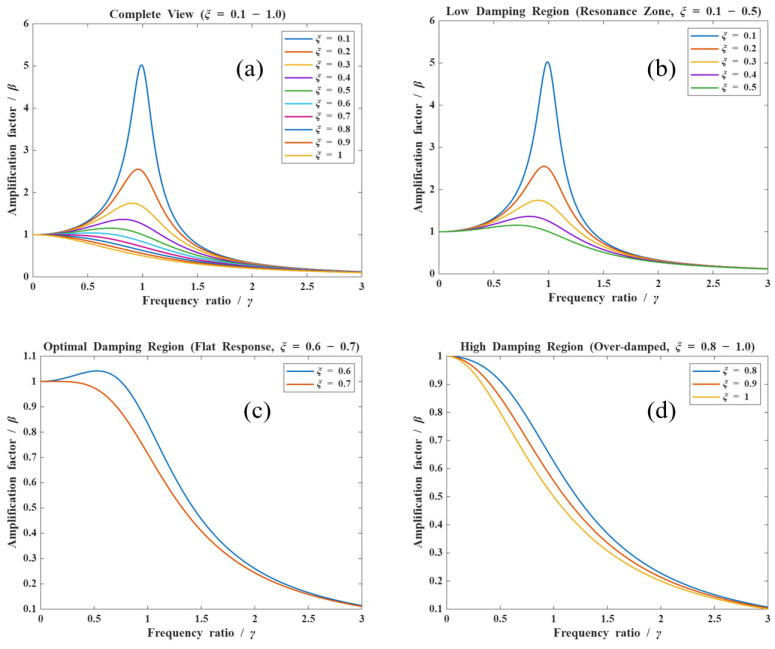
Amplitude–Frequency Characteristic Curve. (**a**) Overall response across all damping ratios (*ξ* = 0.1–1.0); (**b**) Low damping region (resonance zone, *ξ* = 0.1–0.5); (**c**) Optimal damping region (flat response, *ξ* = 0.6–0.7); (**d**) High damping region (over-damped, *ξ* = 0.8–1.0).

The acceleration sensing structure was simulated according to Equation (1), and the simulation results are shown in Figure 2. The flat portion of the curve represents the operating frequency band of the accelerometer.

The relationship between the frequency ratio γ, damping ratio ξ, and amplification factor β of the acceleration sensor was simulated; the results are shown in Figure 2. The amplitude–frequency characteristic curve is relatively flat in the region where the frequency ratio is much smaller than 1; this region is referred to as the flat response region of the acceleration sensor. The wider this flat region, the better the sensor can maintain stability over a broad range of frequency ratios, indicating superior frequency response and overall performance. As the frequency ratio increases, structures with different damping ratios exhibit distinct responses. For sensors with a damping ratio in the range of 0.1–0.5, the amplification factor reaches a peak, indicating that the sensor resonates with the external excitation. For structures with a damping ratio between 0.8 and 1.0, the amplification factor exhibits a decreasing trend, which implies that the system is overdamped, causing frequency lag and an inability to respond promptly to high-speed signals. When the damping ratio is approximately 0.6–0.7, the flat region is widest compared to other damping ratios, and the phase–frequency response curve is nearly linear, enabling the sensor to reproduce the input signal without distortion. The damping ratio ξ of the system is determined by factors such as the sensor’s structure and materials; once the sensor is fabricated, the system’s damping ratio ξ becomes fixed.

According to the FBG sensing principle, the relationship between the relative drift of the center wavelength and axial strain and changes in ambient temperature is as follows:

(2) $$\frac{\Delta \lambda_{B}}{\lambda_{B}}=S_{\varepsilon }\varepsilon +S_{T}\cdot \Delta T=\left(1-P_{e}\right)\varepsilon +\left(\alpha_{n}+\alpha_{\Lambda }\right)\Delta T$$

where *λ_B_* is the center wavelength of the FBG, Δ*λ_B_* is the drift in the center wavelength of the FBG, *S_ε_* is the strain sensitivity coefficient of the FBG, *ε* is the axial micro-strain of the FBG, *S_T_* is the temperature sensitivity coefficient of the FBG, Δ*T* is the change in ambient temperature, *P_e_* is the effective elastic refractive index of the fiber, *α_n_* is the temperature coefficient of the fiber’s refractive index, and *α*_Λ_ is the temperature coefficient of the grating period. In the acceleration measurement experiment, temperature control measures ensure a constant ambient temperature (Δ*T* = 0). the effect of temperature on the FBG wavelength is negligible, and the relative wavelength shift is proportional only to the axial strain. Combining this with the dynamic characteristics of a single-degree-of-freedom vibrating system, we obtain:

(3) $$\frac{\Delta \lambda_{B}}{\lambda_{B}}=\left(1-P_{e}\right)\varepsilon =\frac{\left(1-P_{e}\right)a}{\omega_{0}^{2}L\sqrt{{\left(1-\gamma^{2}\right)}^{2}+{\left(2\xi \gamma \right)}^{2}}}$$

where a denotes acceleration. The acceleration sensitivity *S* of an FBG accelerometer reflects the sensor’s ability to detect vibration acceleration signals. It can be expressed as the change in the FBG’s wavelength when the sensor detects a unit acceleration. From Equation (3), we can derive that:

(4) $$S=\frac{\left(1-P_{e}\right)\lambda_{B}}{\omega_{0}^{2}L\sqrt{{\left(1-\gamma^{2}\right)}^{2}+{\left(2\xi \gamma \right)}^{2}}}$$

When *γ* is much less than 1, the sensitivity of the FBG accelerometer can be expressed as:

(5) $$S=\frac{\left(1-P_{e}\right)\lambda_{B}}{\omega_{0}^{2}L}$$

Although the damping ratio is retained in Equation (1) for generality, it is omitted in the subsequent sensitivity derivation because the impact pulse duration is significantly shorter than the sensor’s natural period. Under this quasi-static condition, energy dissipation via damping is negligible, and the dynamic magnification factor approaches unity.

At this point, the system’s resonant frequency *f*_0_ can be expressed as:

(6) $$f_{0}=\frac{\omega_{0}}{2\pi }=\frac{1}{2\pi }\sqrt{\frac{K}{M}}$$

### 2.2. Working Principle of FBG Acceleration Sensor Based on Double-Sided Symmetrical Inclined Cantilever Beam

**Figure 3:**
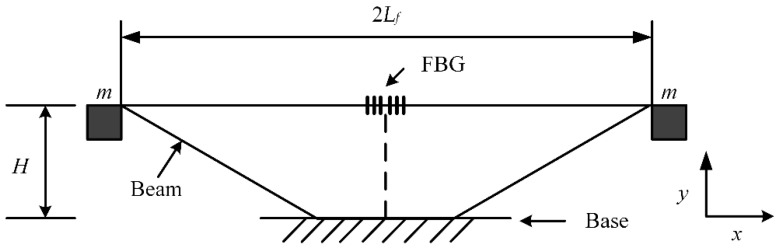
Schematic Diagram of the Acceleration Sensor.

The bilaterally symmetric tilted cantilever beam FBG accelerometer proposed in this paper employs a bilaterally symmetric tilted cantilever beam as the elastic element, with the FBG encapsulated between the two cantilever beams. By utilizing the symmetric deformation of the beams, the design ensures that the FBG experiences only axial strain, thereby preventing damage to the FBG caused by lateral shear forces while enhancing the sensor’s resistance to lateral interference. Its structural model is shown in Figure 3 and consists primarily of a base, bilaterally symmetric tilted cantilever beams, an inertial mass block, and an FBG sensing unit.

The stiffnesses of the optical fiber and the beam are *K*_1_ and *K*_2_, respectively:

(7) $$K_{1}=\frac{E_{1}A}{L_{f}},K_{2}=\frac{E_{2}bt^{3}}{4L^{3}}$$

In the equation: *E*_1_ and *E*_2_ are the elastic moduli of the optical fiber and the cantilever beam, respectively; *A* is the cross-sectional area of the optical fiber; *L_f_* is the effective length of the optical fiber; and *b*, *t*, and *L* are the width, thickness, and total length of the cantilever beam, respectively.

Since the double-sided cantilever beams are symmetrically inclined, the equivalent total stiffness of the system is determined jointly by the axial stiffness of the optical fiber and the bending stiffness of the cantilever beams. Taking into account the inclination angle and geometric structure of the beams, the expression for the equivalent total stiffness *K* is [12]:

(8) $$K=\frac{4}{3}\left(K_{1}\frac{H^{2}}{L^{2}}+K_{2}\right)$$

where *H* represents the geometric component of the cantilever beam in the *Y* direction, reflecting the degree of the beam’s tilt. By substituting the equivalent total stiffness into the theoretical model for sensitivity and resonant frequency, and taking into account the structural characteristics of a bilaterally symmetrical tilted cantilever beam while neglecting the effects of damping and the frequency ratio (γ ≪ 1 in the operating range), a simplified formula for calculating the sensitivity of the FBG accelerometer in this structure is derived:

(9) $$S=\frac{\left(1-P_{e}\right)\lambda_{0}}{\sqrt{3}{\left(2\pi f_{0}\right)}^{2}L_{f}}$$

*λ*_0_ represents the initial center wavelength of the FBG when it is not vibrating. This formula establishes a quantitative relationship between the sensor’s sensitivity and the effective refractive index of the fiber, the initial center wavelength, and the effective length of the fiber, thereby providing a theoretical basis for optimizing the sensor’s structural parameters.

It should be noted that Equation (9) is derived under the quasi-static assumption, which is physically valid for the high-g impact measurement in this work. The impact pulse duration (t_p_ ≈ 0.25 ms) is significantly shorter than the sensor’s natural period (T_0_ = 1/f_0_ ≈ 0.75 ms). Under this condition, the system does not have sufficient time to develop a dynamic resonant response, and the strain is governed by the instantaneous inertial force rather than dynamic amplification. Furthermore, although the impact contains high-frequency components, the dynamic magnification factor approaches unity when the frequency ratio is either much less than 1 or much greater than 1, and the small damping ratio further suppresses oscillatory deviations. Therefore, the omission of the damping and frequency-ratio terms in Equation (9) is justified for calculating the peak strain under 500 g impact conditions.

## 3. Design of FBG Acceleration Sensor

### 3.1. Parameter Design of the Sensor

**Figure 4:**
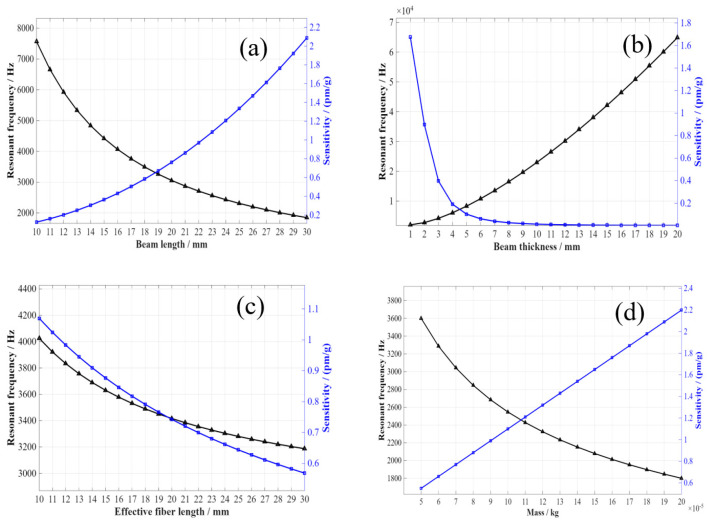
Influence of Structural Parameters on Resonant Frequency and Sensitivity. (**a**) Effect of arm length (L); (**b**) Effect of arm thickness (t); (**c**) Effect of effective optical fiber length (Lf); (**d**) Effect of proof mass (M).

The resonant frequency *f*_0_ and sensitivity *S* are two important parameters of an accelerometer. The resonant frequency determines the sensor’s operating frequency band; the higher the resonant frequency of an FBG accelerometer, the wider its operating frequency band and the flatter its operating range. The resonant frequency *f*_0_ and sensitivity *S* of an FBG accelerometer are determined by the sensor’s structural parameters and are closely related to the elastic moduli of the rigid beam and optical fiber, as well as the beam’s length, width, and thickness, the effective length of the optical fiber, and the masses of the beam and the mass block. Based on the established dynamic model of the FBG-mounted cantilever beam system, the curves in Figure 4 were obtained through a controlled-variable numerical simulation: for each subfigure, one structural parameter was varied within a specified range while all other parameters were kept constant, and the corresponding resonant frequency and sensitivity were calculated using the derived formulas.

To achieve an FBG accelerometer with a higher resonant frequency while ensuring ease of installation and cost-effectiveness, this paper employs PPA-CF material for both the rigid beam and the encapsulation. PPA-CF has an elastic modulus of 11.8 GPa and a density of 1.25 g/cm^3^. The elastic modulus of single-mode quartz optical fiber is 69.9 GPa, and the center wavelength of the FBG is 1549.9 nm when there is no vibration. To facilitate sensor fabrication and packaging, the beam width is set to 2 mm. To achieve a high resonant frequency, the following analysis and research were conducted on the sensor’s structural parameters. Based on the above parameters, the sensor structure was simulated to investigate the sensor parameters that yield optimal performance. The simulation results are shown in Figure 3. Figure 4a illustrates the relationship between beam length and resonant frequency and sensitivity. As shown in the figure, the resonant frequency decreases as the beam length increases, while the sensitivity increases with increasing beam length. To achieve a higher resonant frequency, L = 21.15 mm was selected. Figure 4b illustrates the relationship between beam thickness and the resonant frequency and sensitivity. As shown in the figure, the resonant frequency decreases as the beam thickness increases, while the sensitivity increases. To achieve a higher resonant frequency, t = 2 mm is selected. Figure 4c shows the relationship between the effective fiber length and the resonant frequency and sensitivity. As shown in the figure, as the effective length of the optical fiber increases, the resonant frequency decreases, and sensitivity decreases accordingly. To achieve a higher resonant frequency, L_f_ was set to 24.32 mm. Figure 4d shows the relationship between the mass of the mass block and the resonant frequency and sensitivity. As shown in the figure, the resonant frequency decreases as the mass of the mass block increases, and the sensitivity decreases as the beam length increases. To achieve a higher resonant frequency, t = 8 × 10^−5^ kg is selected. The final parameters of the FBG accelerometer are as follows: the effective elastic modulus of the optical fiber is 0.22, the center wavelength is 1550 nm, the effective length of the optical fiber is 24.32 mm, the geometric dimension of the beam in the Y-direction is 10.58 mm, and the mass of the mass block is 8 × 10^−5^ kg; Regarding material parameters, the elastic modulus of the optical fiber is 69.9 GPa, and its cross-sectional area is 1.227 × 10^−8^ m^2^. The elastic modulus of the beam material (PPA-CF) is 11.8 GPa, the beam width is 4 mm, the total beam length is 21.15 mm, and the beam thickness is 2 mm. These parameters are collectively used to calculate key performance metrics of the system, such as equivalent stiffness, resonant frequency, and sensitivity.

### 3.2. Modal Analysis of FBG Acceleration Sensor

**Figure 5:**
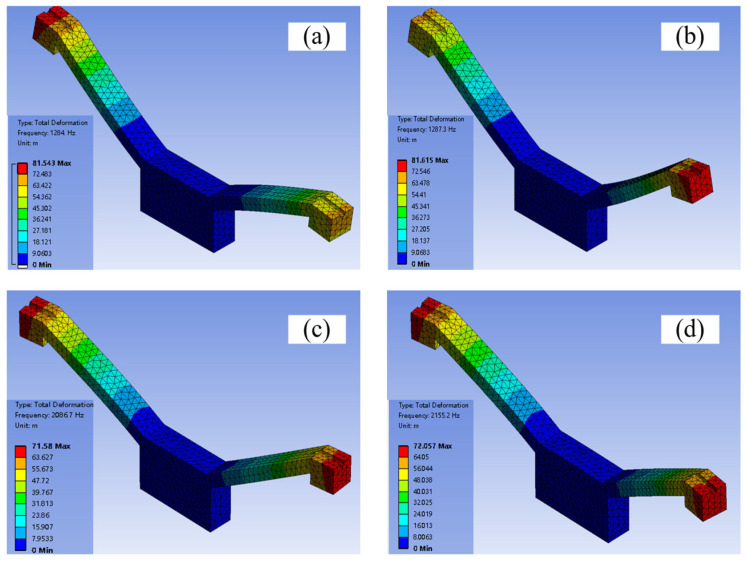
Mode shapes of the FBG acceleration sensor at various orders. (**a**) First-order mode shape, (**b**) Second-order mode shape, (**c**) Third-order mode shape, (**d**) Fourth-order mode shape.

To visually illustrate the modal characteristics of the sensor, a modal analysis of the sensor structure was performed using ANSYS 2022r1software; the first four modal modes are shown in Figure 5.

Figure 5a shows that the first-order modal frequency of the sensor is 1284.0 Hz. The mode shape is characterized by a double-sided cantilever beam driving a mass block to oscillate up and down in phase around the fixed end along the Z-axis, exhibiting a basic bending vibration pattern. Figure 5b shows that the sensor’s second-order modal frequency is 1287.3 Hz. The first two modal frequencies are 1284.0 Hz and 1287.3 Hz, respectively. Although they appear close, the corresponding mode shapes exhibit distinct orthogonal characteristics, ensuring that the dominant vibration remains along the axial direction without significant coupling that would degrade the pure axial strain transfer. The mode shape consists of a double-sided cantilever beam driving a mass block to oscillate up and down in the Z-axis direction around the fixed end in opposite phases—that is, when one side moves upward, the other moves downward—exhibiting first-order torsional vibration characteristics. Figure 5c shows that the third-order modal frequency of the sensor is 2086.7 Hz, with the mode shape characterized by the mass block rotating about the sensor’s central axis in the YZ plane, while the cantilever beam undergoes more complex bending deformation. Figure 5d shows the sensor’s fourth-order modal frequency of 2155.2 Hz, with the mode shape characterized by localized high-order bending vibrations in the mass-cantilever system, resulting in a significant increase in mode shape complexity.

The modal analysis results confirm that the first-order mode at 1284.0 Hz is characterized by pure axial translation of the mass block along the Z-axis. Crucially, the double-sided symmetrical inclined structure ensures that any transverse bending moments acting on the left and right beams cancel each other out. This kinematic constraint guarantees that the FBG, encapsulated between the beams, is subjected exclusively to axial tensile or compressive strain, effectively eliminating parasitic transverse shear strain components.

### 3.3. Stress and Deformation Analysis of FBG Acceleration Sensor

The sensor elastomer was analyzed using the ANSYS Static Stress Analysis module. The base of the sensor elastomer was fixed, and a downward acceleration of 500 times the acceleration due to gravity was applied to the upper surface of the mass block. The results of the overall deformation analysis are shown in the figure. The analysis results indicate that under the action of gravitational acceleration, the sensor elastic body undergoes deformation in the y-axis direction, with a maximum deformation of 3.59 × 10^−6^ m. This value is far smaller than the vibration amplitude of the mass block when subjected to external acceleration and will not affect the normal acceleration sensing function of the FBG accelerometer.

**Figure 6:**
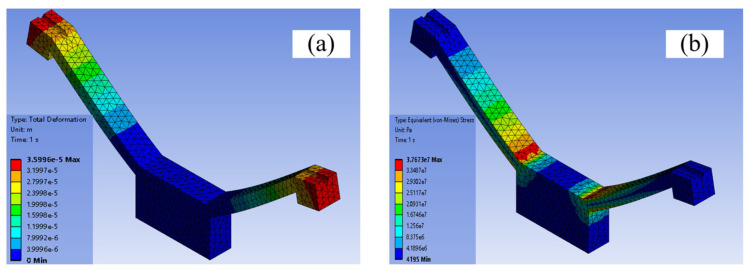
Stress and deformation analysis of the FBG acceleration sensor: (**a**) deformation analysis diagram; (**b**) stress analysis diagram.

Further quantitative validation is provided by the static stress analysis under 500 g acceleration. As shown in Figure 6b, the maximum stress is concentrated at the beam-root junction with a magnitude of approximately 3.7 MPa. This value is significantly lower than the yield strength of the PPA-CF material, indicating that the structure operates in the elastic region without plastic deformation. More importantly, the stress distribution is uniformly aligned along the axial direction of the beam, confirming the absence of significant shear stress concentrations that would otherwise corrupt the FBG wavelength shift signal.

## 4. Experiments

### 4.1. Experimental Equipment

**Figure 7:**
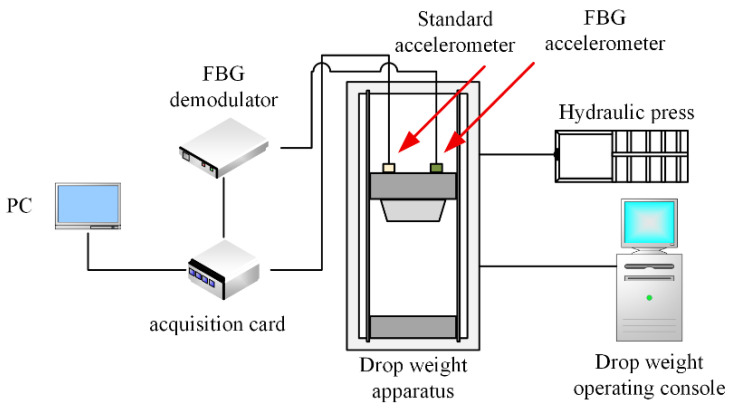
Schematic diagram of experimental equipment.

**Figure 8:**
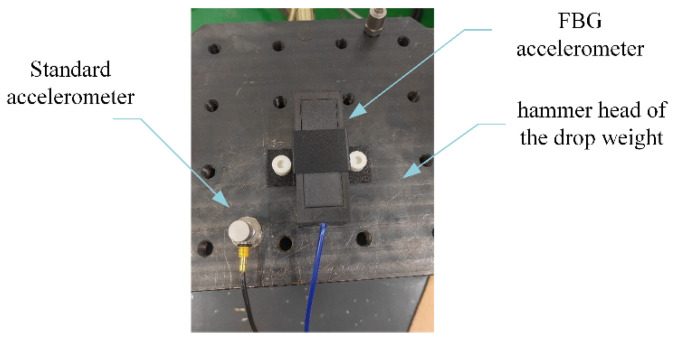
Physical photograph of sensor placement.

To comprehensively calibrate the dynamic performance of FBG accelerometers, a drop-weight dynamic calibration system was established. As shown in Figure 7, the system consists of a drop-weight test bench, a drop-weight apparatus, a reference accelerometer, the accelerometer under test, an FBG demodulator, a data acquisition card, and a PC-based data processing system, thereby establishing a complete test workflow comprising “impact excitation–synchronous detection–signal conversion–data processing.” The hydraulically driven drop-weight test stand controls the drop-weight assembly to generate controllable impact accelerations ranging from 0 to 1000 g, providing repeatable impact excitation for the sensors. The FBG accelerometer is rigidly mounted using a specialized fixture, while the standard accelerometer is attached to the same mounting base on the drop-weight impact platform via a strong magnet, as shown in Figure 8. This ensures that both sensors experience the same impact acceleration simultaneously, with the standard accelerometer serving as the reference for calibration. The FBG demodulator converts the sensor’s wavelength shift signal into an electrical signal, with a voltage change of 1000 mV corresponding to a wavelength shift of 1 nm. This signal is synchronously acquired with the output signal from the standard accelerometer via a data acquisition card, ensuring complete capture of high-frequency impact signals; Finally, dedicated PC software performs subsequent processing, including signal filtering, linear fitting, sensitivity calculation, and linearity analysis, enabling systematic testing of the sensor’s amplitude–frequency characteristics and linear response characteristics.

### 4.2. Impact Response Test

**Figure 9:**
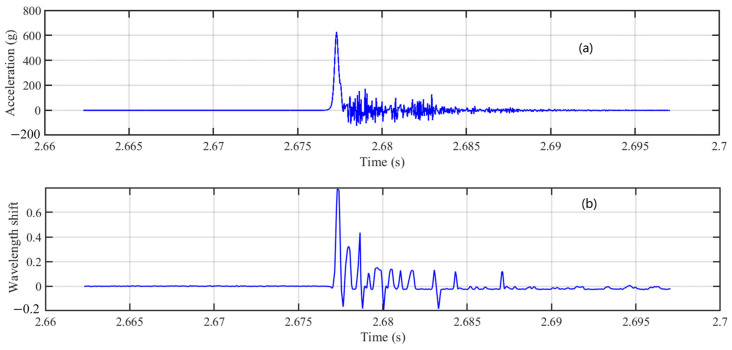
Time-domain waveforms: (**a**) time-domain waveform of the standard acceleration sensor; (**b**) time-domain waveform of the FBG acceleration sensor.

First, the time-domain waveform characteristics of the FBG sensor and the standard accelerometer under impact were observed. The measurement results are shown in Figure 9. Figure 9a presents the time-domain waveform of the standard accelerometer, and Figure 9b shows that of the FBG accelerometer under test. The waveform of the FBG accelerometer starts to rise at 2.67705 s, reaches its peak at 2.6773 s, and ends the impact at 2.67755 s. The waveform of the standard accelerometer begins to rise at 2.67703 s, peaks at 2.6773 s (the acceleration is 626.7 g), and finishes the impact at 2.6777 s. The maximum time delay between the two accelerometers does not exceed 0.15 ms. In the latter half after the impact, both accelerometers exhibit waveform fluctuations, which arise from the resonance of the elastic element. In practical engineering, sensor performance is generally evaluated by comparing the first effective impact peak.

The frequency response of the sensor is calculated according to the following formula:

$$f_{0}=\frac{1}{t_{\max}-t_{start}}\cdot \frac{1}{3}$$

In the formula, *t*_max_ represents the time at which the pulse reaches its maximum value, and t*_start_* is the start time of the pulse. Consequently, the frequency response of the FBG accelerometer is obtained as 1333 Hz, which is in good agreement with the simulation result of 1284 Hz. The overall sensor specifications are shown in Table 1.

### 4.3. Linear Response Characteristics 

Linear response characteristics are a key metric for evaluating the measurement accuracy of an accelerometer; they directly reflect the degree of linear correlation between the sensor’s output signal and the input acceleration, and the sensor’s actual acceleration sensitivity can be calculated from the results of linear fitting. To accurately test the linear response characteristics and actual sensitivity of the designed FBG accelerometer, the laboratory ambient temperature was maintained at a constant level throughout the experiment. This effectively eliminated the interference of temperature drift on the FBG’s central wavelength, ensuring that the experimental data reflected only the correlation between acceleration and wavelength drift. The test generated eight sets of graded impact acceleration stimuli within the range of 400 g to 600 g by controlling the drop height of the falling hammer in stages. To ensure the reliability and repeatability of the test data, each set of acceleration stimuli was tested five times. The arithmetic mean of the five test results was taken as the valid data corresponding to that set of acceleration, ultimately yielding eight sets of valid acceleration-voltage correspondence data.

The acceleration sensitivity of a sensor is defined as the change in the central wavelength of the FBG per unit acceleration, measured in pm/g. Considering the wavelength-to-voltage conversion relationship of the demodulator and the physical significance of the linear fit slope—where the slope represents the change in acceleration corresponding to a unit change in voltage—the actual acceleration sensitivity of the sensor is the reciprocal of the fit slope. Thus, the conversion is performed by calculating the ratio of the change in voltage to the change in acceleration. The acceleration sensitivity of 1.183 for this FBG acceleration sensor.

**Figure 10:**
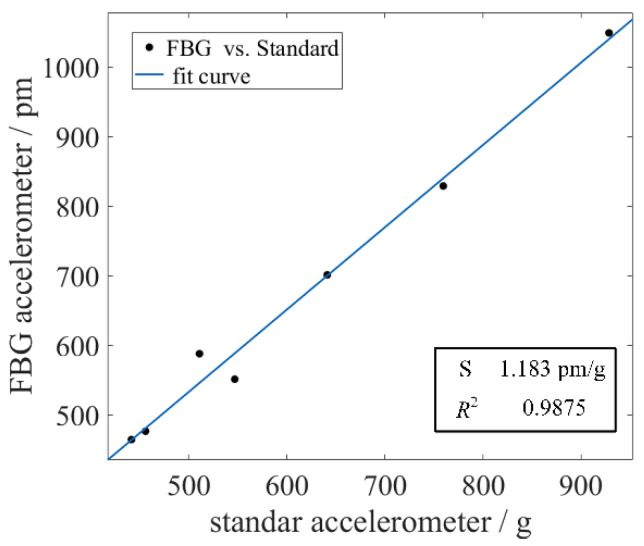
Sensitivity fitting curve.

A comprehensive analysis of the linear response test results is shown in Figure 10, and lead below conclusion: First, both the coefficient of determination for the experimental data and the adjusted coefficient of determination are close to 1, and the *R*^2^ is as high as 0.9875. This fully demonstrates that within the impact acceleration range of 400 g to 600 g, the output voltage of this FBG accelerometer exhibits a good linear response relationship with the input acceleration. The sensor has excellent linearity and can meet the basic requirements for quantitative measurement of high-frequency impact acceleration. Second, the sensor’s actual measured sensitivity is 1.183 pm/g. This value primarily stems from positioning errors during the bonding of the optical fiber to the cantilever beam, minor dimensional errors in the cantilever beam’s fabrication, and slight mechanical fluctuations during the drop-weight impact process; there are no systematic errors. Third, although this sensitivity value differs somewhat from that of low-frequency FBG accelerometers, it represents a measurement sensitivity of practical engineering value for high-frequency FBG accelerometers with resonance frequencies exceeding 1000 Hz. Combined with its wide bandwidth characteristics, it can meet the detection requirements for high-frequency impact acceleration in fields such as aerospace and impact dynamics.

## 5. Conclusions

To address the need for high-range, high-frequency impact acceleration measurement, this paper designs and develops an FBG accelerometer featuring a bilaterally symmetric tilted cantilever beam structure. By combining theoretical modeling, simulation optimization, and experimental testing, the design, fabrication, and performance characterization of the sensor were systematically completed. The main research findings are as follows:

(1) A structure for a double-sided symmetrical tilted cantilever beam FBG accelerometer was proposed. Based on the principles of inertia and FBG sensing, theoretical models for the sensor’s sensitivity and resonant frequency were derived, clarifying the inverse-square relationship between the two. Combining the geometric characteristics of this structure, simplified calculation formulas for equivalent stiffness and sensitivity were established, providing a theoretical basis for optimizing the structural parameters of high-frequency FBG accelerometers.
(2) With a resonance frequency greater than 1333 Hz as the core design objective, numerical simulations were conducted to analyze the influence of cantilever length, thickness, effective fiber length, and mass block mass on sensor performance, thereby determining the optimal structural parameters. Finite element simulation results indicate that the sensor’s first-order natural frequency is 1284 Hz, with a measured sensitivity of approximately 1.183 pm/g. The vibration mode is axial translational motion of the mass block, ensuring that the FBG is subjected only to axial strain without additional stress, thereby validating the rationality of the structural design.
(3) With a resonance frequency exceeding 1333 Hz, this sensor achieves a measurement range of over 500 g and a practical sensitivity of approximately 1.183 pm/g. It balances high-frequency performance with measurement accuracy, providing a novel and reliable technical solution for high-frequency impact acceleration monitoring in fields such as aerospace and impact dynamics.

## Figures and Tables

**Table 1:** Sensor performance specifications.

Sensor Type	FBG Accelerometer	Standard Accelerometer
Impact start time (s)	2.67705	2.67703
Impact peak time (s)	2.67730	2.67730
Impact end time (s)	2.67755	2.67770
Impact pulse duration (ms)	0.50	0.67
Frequency response (Hz)	1333 Hz	1234 Hz
Maximum time delay error (ms)	0.15	0.15

## Data Availability

The original data presented in this study are available from the corresponding author upon reasonable request.
